# Clinical outcome for patients with dedifferentiated chondrosarcoma: a report of 9 cases at a single institute

**DOI:** 10.1186/1749-799X-7-38

**Published:** 2012-12-10

**Authors:** Kazuya Yokota, Akio Sakamoto, Yoshihiro Matsumoto, Shuichi Matsuda, Katsumi Harimaya, Yoshinao Oda, Yukihide Iwamoto

**Affiliations:** 1 Department of Orthopaedic Surgery, Graduate School of Medical Sciences, Kyushu University, Fukuoka, 812-8582, Japan; 2 Department of Anatomic Pathology, Graduate School of Medical Sciences, Kyushu University, Fukuoka, 812-8582, Japan

## Abstract

**Background:**

Dedifferentiated chondrosarcomas consist of two distinguishable components: low-grade chondrosarcoma components and high-grade dedifferentiated components.

**Materials and methods:**

Nine cases (4 males, 5 females) of dedifferentiated chondrosarcoma were treated in our institute. The average age was 58.6 (range, 37–86) years. The tumor location was the long bone in 7 cases (femur, n=5; humerus, n=1; tibia, n=1) and the pelvic bone in 2 cases. The average time from appearance of symptoms to treatment was 9.4 (range, 1–40) months.

**Results and discussion:**

On plain radiographs, matrix mineralization was seen in all 9 cases (100%). Bone destruction was observed in 5 of 9 cases (56%), while pathological fracture was seen in one femur case (11%). Lung metastasis was observed in all cases (initially in 5 cases; during the treatment course in 4 cases). Surgery was performed in 8 cases, with local recurrence occurring in 2 of those cases (time to recurrence, 2 and 10 months). Chemotherapy was administered in 4 cases, but did not result in significant improvement. All 9 cases died of lung metastases, with a median survival time of 10 (range, 3.4-18.8) months. The presence of initial metastasis at diagnosis was a significant unfavorable prognostic factor.

**Conclusion:**

The prognosis of dedifferentiated chondrosarcoma is dismal. With the lack of convincing evidence of the benefit of chemotherapy, complete surgical excision is the initial recommended treatment.

## Background

Dedifferentiated chondrosarcomas consist of two distinguishable components: low-grade chondrosarcoma components and high-grade dedifferentiated components. This group of cancers was first described in 1971 [1]. The dedifferentiated components have varying features, including features of undifferentiated sarcomas, osteosarcomas, angiosarcomas, fibrosarcomas, rhabdomyosarcomas, leiomyosarcomas, and giant cell tumors [2]. Dedifferentiated chondrosarcomas are highly malignant with a very poor prognosis [3,4]. Surgery is the primary treatment modality, and chemotherapy has been adapted for use in selected cases with dedifferentiated chondrosarcoma [2,4,5]. In the current report, a series of dedifferentiated chondrosarcoma cases treated in our institute is examined, with a focus on identification of prognostic factors.

## Materials and methods

Between 1996 and 2010, 9 cases of dedifferentiated chondrosarcoma were treated in our institute. All cases had been referred to our institute by nearby hospitals. Clinical information of these cases is presented in Table 1. The cases included 4 males and 5 females. The mean age at the first procedure was 58.6 years (range, 37 to 86 years). The tumor location was the long bone in 7 cases (femur, n=5; humerus, n=1; tibia, n=1). In the other 2 cases, tumors were located in non-long bone (pelvic bone in both cases). Biopsy was performed in all cases for the purpose of diagnosis. Surgery was performed in 8 cases, while 1 case received palliative care only.

**Table 1:** Clinical information of cases with dedifferentiated chondrosarcoma

**Case**	**Age/Sex**	**Location**	**Term of symptom**	**Extraskeletal extension**	**Chemotherapy**	**Surgical margin**	**Local recurrence (time from surgery)**	**Time to lung metastasis**	**Outcome**
#1	53/F	Femur	9 m	+	-	WR	-	Initial	DOD (3.4 m)
#2	37/F	Tibia	2 m	+	+	WR	+ (10 m)	9 m	DOD (14.1 m)
#3	71/M	Pelvis	24 m	+	-	IR	-	Initial	DOD (5.0 m)
#4	59/M	Humerus	40 m	+	+	WR	+ (2 m)	4 m	DOD (10 m)
#5	54/F	Pelvis	3 m	+	+	MR	-	Initial	DOD (9.1 m)
#6	71/F	Femur	3 m	+	-	WR	-	15 m*	DOD (18.8 m)
#7	44/M	Femur	2 m	+	+	WR	-	10 m	DOD (14.0 m)
#8	73/M	Femur	1 m	+	-	WR	-	Initial	DOD (6.7 m)
#9	86/F	Femur	1 m	+	-	-	NA	Initial	DOD (5.0 m)

F, female; M, male; PD, progressive disease; WR, wide resection; IR, intralesional resection; MR, marginal resection; M, months; NA, not assessed; *, as well as bone metastasis; DOD, died of disease.

Plain radiographs taken at the initial visit to our institute were assessed. They were evaluated with respect to matrix mineralization, bone destruction, and the presence of fracture. The presence of extraskeletal extension was assessed by magnetic resonance imaging (MRI). Surgical treatment was performed by either resection alone or resection followed by implantation of an endoprosthesis. Chemotherapy was administered to select cases.

### Statistical analysis

The survival estimates were determined by Kaplan-Meier analysis, while the survival differences according to clinical parameters (lung metastasis, local recurrence, and location) were evaluated by the log-rank test. A *P* value of less than 0.05 was considered to indicate statistical significance.

## Results

Time from the appearance of symptoms to treatment ranged from 1 to 40 months, with an average of 9.4 months and a median of 3 months. All 9 cases presented with pain (100%). A palpable mass was detected in 3 of the 9 cases (33%), including 1 humerus case and 2 femur cases. Numbness was observed in 1 of the 9 cases (11%), a femur case (Table 2).

**Table 2:** Summary of clinical symptoms and images in cases of dedifferentiated chondrosarcoma

**Age (year)**	**Sex (M/F)**	**Location (long bone /trunk)**	**Pain**	**Palpable mass**	**Numbness**	**Matrix mineralization**	**Bone destruction**	**Fracture**	**Extra-osseous extension**
58.6	4/5	7/2	9/9	3/9	1/9	9/9	5/9	1/9	6/9
			(100)	(33%)	(11%)	(100%)	(56%)	(11%)	(67%)

M, male; F, female.

On plain radiographs, matrix mineralization was observed in all 9 cases (100%). Bone destruction was detectable in 5 of the 9 cases (56%). Pathological fracture was observed in 1 femur case (11%). On MRI, extraskeletal extension was seen in 6 of the 9 cases (67%). All of the cases with bone destruction on plain radiographs had extraskeletal extension on MRI (Table 2).

Eight of the nine cases were managed with surgery (89%), while the other one case received non-surgical palliative care only. This latter patient was an 86-year-old woman with dedifferentiated chondrosarcoma in the femur who refused to undergo amputation. Among the 8 cases who underwent surgery, 6 cases were given a wide surgical margin (75%), 1 case was given a marginal margin (13%), and 1 case underwent intralesional resection (13%). In the long bone cases, wide resection was performed in all 6 cases. In contrast, in the pelvic bone cases, 1 case was given a marginal margin, while the other case underwent intralesional resection (Table 1).

Chemotherapy was given to 4 patients following surgical tumor resection. The chemotherapy regimen consisted of adriamycin, ifosfamide, cisplatin, and methotrexate.

Lung metastasis was observed in all cases (initially in 5 cases; during the treatment course in 4 cases). In one case, bone metastasis was seen in addition to the lung metastasis. Time to lung metastasis ranged from 4 to 15 months, with an average of 9.5 months. Local recurrence following resection occurred in 2 of the 8 surgery cases, at 2 and 10 months. Both of these local recurrence cases had undergone amputation (Table 1).

**Figure 1:**
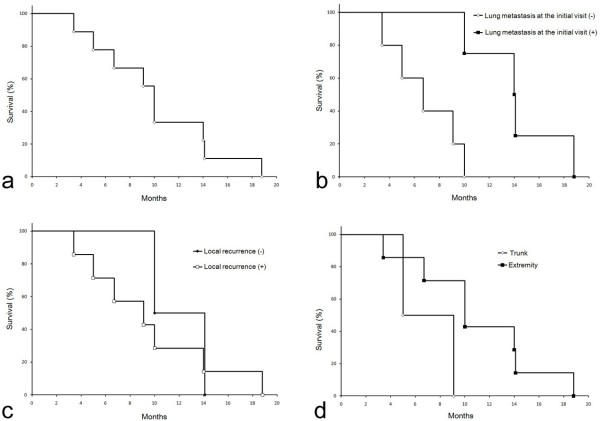
**(a) Kaplan-Meier survival curve for all 9 cases with dedifferentiated chondrosarcoma, showing a mean survival time of 10 months.** (**b**) Overall survival curves comparing cases with lung metastasis at the initial visit and those without; the difference is significant. (**c**) Overall survival curves comparing cases with local recurrence and those without. (**d**) Overall survival curves comparing cases in the trunk and those in the extremities.

The median survival time was 10 months, with a mean survival time of 10.1 months, ranging from 3.4 to 18.8 months (Table 1, Figure 1a). All 9 cases died of lung metastases. The presence of metastasis at diagnosis was a significant unfavorable prognostic factor (Figure 1b). The median survival time was 6.7 months in cases with metastases at diagnosis (mean survival time, 5.8 months) and 14 months in cases without metastases at diagnosis (mean survival time, 14.2 months). No significant difference in survival based on local recurrence was observed; the mean survival time was 12.1 months in cases with local recurrence and 9.5 months in cases without local recurrence (Figure 1c). Patients with trunk tumors tended to have shorter survival (mean, 7.1 months; median, 5 months) than patients with extremity tumors (mean, 10.3 months; median, 10 months) (Figure 1d). Neither gender nor age were significant factors. No significant difference in survival was noted between cases that received chemotherapy and those who did not. However, the median survival time was 11.8 months for the 4 cases who received chemotherapy (mean survival time, 12 months) while it was 9.1 months for the cases who did not receive chemotherapy (mean survival time, 7.8 months).

## Discussion

Dedifferentiated chondrosarcoma is known to have a poor prognosis. Median survival has been as short as 6 months, and 5-year survival rates may be as low as 10% to 13%. Patients rarely survive for more than 2 years [3,4]. Consistent with the reported unfavorable prognosis, all current cases died of lung metastases, with a median survival time of only 10 months (range, 3.4 to 18.8 months). In two very recent reports, the median survival time was 7.5 months [6] and 1.4 year [5], and the overall 5-year survival rate was 7.1% [6] and 24% [5], respectively. The prognosis of dedifferentiated chondrosarcoma has shown a slow improvement trend over time, presumably due to earlier diagnosis and improved staging and treatment [5].

Metastasis, especially to the lung, is the most important treatment problem in patients with dedifferentiated chondrosarcoma [5-7]. In the current study, the presence of metastases at diagnosis was a significant unfavorable prognostic factor. Chemotherapy is an option for control of metastatic lesions. However, the value of chemotherapy for cases with dedifferentiated chondrosarcoma has not been supported [2,4]. In addition to the lack of convincing evidence of the benefit of chemotherapy, the toxicity of chemotherapy experienced by older patients with dedifferentiated chondrosarcoma generally rules it out as a standard treatment [5]. However, in a previous report, 2 out of 13 patients with dedifferentiated chondrosarcoma experienced a good response to chemotherapy, demonstrating more than 90% necrosis [5]. These results suggest a possible benefit of chemotherapy for some patients with dedifferentiated chondrosarcoma.

The prognosis of patients with dedifferentiated chondrosarcoma can be improved by an accurate preoperative diagnosis [8]. Characteristically on plain radiographs, a combined pattern composed of the aggressive parts of dedifferentiated chondrosarcoma components and the less aggressive parts of well-differentiated chondrosarcoma components suggests dedifferentiated chondrosarcoma [8,9]. Reflecting the aggressive nature of dedifferentiated chondrosarcoma, osteolytic lesions associated with cortical destruction have been reported in the majority of cases on plain radiographs [9]. In the current study, bone destruction was present in about half of the cases on plain radiographs, and all of these cases demonstrated extraskeletal extension on MRI. The presence of matrix mineralization, indicative of a cartilaginous tumor, accompanied by bone destruction of an aggressive nature may suggest a diagnosis of dedifferentiated chondrosarcoma based on plain radiographs.

It is important to distinguish between conventional chondrosarcoma and dedifferentiated chondrosarcoma, considering the greatly different prognosis [2]. A pathological diagnosis would be inaccurate if the biopsy sample contained just one component of dedifferentiated chondrosarcoma. Therefore, a carefully planned biopsy based upon MRI, assessing each component of dedifferentiated chondrosarcoma, is essential. Even if only one component—either a conventional chondrosarcoma component or a dedifferentiated component—is detected in the pathological sample, possible dedifferentiated chondrosarcoma should be considered in cases in whom plain radiographs show matrix mineralization with a destructive aggressive feature.

Local control by surgery does not appear to be related to prognosis [5-7]. This observation seems to emphasize the importance of not only local control, but also control regarding metastasis, which is directly associated with the prognosis. However, surgical treatment for local control is still an important procedure for dedifferentiated chondrosarcoma. Local recurrence has been reported in up to 50% of cases after excision, with much better local control when there is adequate, or wide, resection [3-5]. In the current study, local recurrence was seen in 2 out of 6 cases treated with wide resection, including 1 case in the tibia and 1 case in the humerus. Since the tibia and humerus are close to essential vessels, the creation of an adequate surgical margin intended to prevent local control may be difficult.

Regarding the pathogenesis of dedifferentiated chondrosarcoma, no structural or numerical chromosomal aberrations that are highly specific for dedifferentiated chondrosarcomas have been identified. However, some evidence has suggested clustering of breakpoints in specific regions of 6q13-22 and 9p21-24 in dedifferentiated chondrosarcoma [10]. High-grade dedifferentiated components have been shown to have a higher malignant potential than low-grade chondrosarcoma components, with increased proliferation as demonstrated by expression of Ki-67 and proliferating cell nuclear antigen [11]. The plasminogen activator system is a key regulator of invasion and tumor angiogenesis. Plasminogen activator inhibitor 1 (PAI-1) acts as an inhibitor of tissue-type plasminogen activator (tPA) and urokinase-type plasminogen activator (uPA) [12]. In dedifferentiated chondrosarcoma, high-grade dedifferentiated components display diffuse coexpression of t-PA, u-PA, and PAI-1 [13]. Matrix metalloproteinases (MMPs) are a family of zinc-dependent endopeptidases that are principally involved in the breakdown of the extracellular matrix, as well as in tumor angiogenesis [14]. Upregulation of MMP2 and MT1-MMP has been reported in high-grade malignant cartilaginous tumors, as well as in the high-grade dedifferentiated component of dedifferentiated chondrosarcoma [15].

## Conclusion

The current study describes a series of dedifferentiated chondrosarcoma cases treated in our institute. The presence of initial metastasis at diagnosis was a significant unfavorable prognostic factor. The prognosis of dedifferentiated chondrosarcoma remains dismal. With the lack of convincing evidence of the benefit of chemotherapy for dedifferentiated chondrosarcoma, including what constitutes standard treatment, its use in this patient population remains controversial. Complete surgical excision should be the initial treatment for patients with a dedifferentiated chondrosarcoma, with chemotherapy reserved for palliative purposes.

## Abbreviations

MRI: Magnetic resonance imaging.

## Competing interests

There are no competing interests.

## Authors’ contributions

KY and AS drafted the manuscript. KY, AS, YM, SM, and KH administered the treatment. KY, AS, YM, and YO participated in the design of the study. YI conceived of the study, and participated in its design and coordination and helped to draft the manuscript. All authors read and approved the final manuscript.
